# Correlation between the expression of vegf and survival in osteosarcoma

**DOI:** 10.1590/1413-78522014220500978

**Published:** 2014

**Authors:** André Mathias Baptista, André Ferrari De França Camargo, Renée Zon Filippi, Cláudia Regina Gomes Cardim Mendes De Oliveira, Raymundo Soares De Azevedo, Olavo Pires De Camargo

**Affiliations:** 1 Universidade de São Paulo, Faculdade de Medicina, Hospital das Clínicas, São Paulo, SP, Brazil, Institute of Orthopedics, Hospital das Clínicas da Faculdade de Medicina da Universidade de São Paulo, São Paulo, SP, Brazil; 2 Universidade de São Paulo, Faculdade de Medicina, Department of Pathological Anatomy, São Paulo, SP, Brazil, Department of Pathological Anatomy, Faculdade de Medicina da Universidade de São Paulo, São Paulo, SP, Brazil

**Keywords:** Osteosarcoma, Prognosis, Neovascularization, pathologic

## Abstract

**Objective:**

To present a series of 50 consecutive patients with non-metastatic extremity osteosarcoma, and attempt to correlate expression of the vascular endothelial growth factor (VEGF) protein in biopsy tissue to their prognosis regarding overall survival, disease-free survival and local recurrence.

**Methods:**

Fifty cases of non-metastatic osteosarcoma of the extremities treated between 1986 and 2006 at Instituto de Ortopedia e Traumatologia da Universidade de São Paulo, São Paulo, Brasil, were evaluated regarding expression of the VEGF protein. There were 19 females and 31 males. The mean age was 16 years old (range 5-28 years old) and the mean follow-up was 60.6 months (range 25-167 months). The variables studied were age, gender, anatomic location, type of surgery, surgical margins, tumor size, post chemotherapy necrosis, local recurrence, pulmonary metastasis and death.

**Results:**

Thirty-six patients showed VEGF expression on 30% or less cells (low), and the remaining 14 cases had VEGF expression above 30% (high). Among the 36 patients with low VEGF expression, nine developed pulmonary metastasis and four died (11.1%). Among the 14 patients with high VEGF expression, six developed pulmonary metastasis and three died (21.4%).

**Conclusion:**

There was no statistically significant correlation between the expression of VEGF and any of the variables studied. *Level of Evidence IV, Therapeutic Study. *

## INTRODUCTION

Osteosarcoma treatment changed dramatically in the 80's with the use of multiagent chemotherapy. Before that, osteosarcoma patients were treated only with limb amputation, when feasible, and experienced a survival rate around 15% in 5 years. With the implementation of postoperative chemotherapy, authors realized that the survival time changed considerably. Stimulated by the brilliant results, preoperative chemotherapy was then suggested in order to try to preserve the affected limb.^1^ Lots of papers confirmed the excellent results of mutiagent chemotherapy, which usually included high-dose methotrexate and doxorubicin, and the 5-year survival rates jumped from 15% to around 70% in nonmetastatic patients. Surgery also developed considerably and limb-preserving surgeries, which were the exception before preoperative chemotherapy, became the rule. Amputation rates dropped from 100% to around 20%.

However, even with the huge advancements in surgical techniques, about 30% of the patients still develop metastatic disease and perish along the postoperative period. Efforts have been made in the last two decades in attempt to improve the osteosarcoma survival rate. Changes in the drugs, their number, their doses and administration schemes did not have impact on survival. Regarding chemotherapy, we are still in the same situation as 20 years ago.

With that situation in mind, other paths are being tried in order to advance in the osteosarcoma survival rates. One of most promising field is the research concerning angiogenesis. No solid tumor grows over 2mm without angiogenesis because cells must be within a certain distance of a capillary vessel in order to survive. Theoretically, if tumor angiogenesis could be suppressed, the tumor would not grow over 2mm and thus not metastasize and kill the patient.

One of the most potent angiogenic factors is the vascular endothelial growth factor (VEGF). It is a dimeric glicoprotein of wieght around 36-46 kilodaltons (KDa), which acts promoting angiogenesis and vascular permeability.

VEGF levels been tested as prognostic factor in the most frequent cancers, such as breast, prostate, colorectal, lung, renal cell, glioblastoma and ovary. In 1999, Lee *et al.* 2 were the first to try to establish VEGF expression as a prognostic factor for survival in osteosarcoma patients.

Humanized anti-VEGF monoclonal antibodies, like bevacizumab (Avastin^(r))^, which was approved by the FDA in February 2004, or ranibizumab (Lucentis^(r))^, have proven their effectiveness in some cancers, but still not in osteosarcoma.

The objective of this study is to present a series of 50 consecutive nonmetastatic extremity osteosarcoma patients, and try to correlate the VEGF expression in their biopsy tissue to their prognosis regarding overall survival, disease-free survival and local recurrence.

## MATERIAL AND METHODS

This study was approved by the Ethics Committee (0016/2007).

All osteosarcoma patients treated at the University of São Paulo Medical School Hospital das Clínicas had their charts reviewed from 1986 to 2006.

Three inclusion criteria were defined:1) Primary high grade central osteosarcoma, located in the appendicular skeleton, nonmetastatic at diagnosis; 2) Minimum 24 months of follow-up; 3) Complete data, including biopsy paraffin embedded biopsy tissue.

Among the 195 patients with osteosarcoma treated in the period, 50 filled the above criteria. (Table 1) On these 50 charts, the following data was extracted: Register number: Name; Age at diagnosis; Gender; Anatomic location; Biopsy date; Surgery date; Type of surgery; Microscopic surgical margins; Tumor size; Post CT necrosis; Local recurrence; Distant metastasis; Follow-up period; Last oncologic status.

**Table 1:** Patients data.

**Case #**	**Age**	**Gender**	**Surgery**	**Final status**	**OS**	**DFS**	**Local recurrence**	**Metastasis**	**Death**
1	16	F	05/05/89	NED 26/08/2002	159	159	-	-	-
2	13	M	30/11/89	DOD 20/04/1996	77	44	44	47	77
3	16	M	09/04/90	AWD 09/03/1999	107	98	98	-	-
4	19	M	03/04/91	DOD 03/10/1995	42	26	-	26	42
5	17	M	24/10/91	DOD 08/02/1998	76	17	17	22	76
6	15	F	22/06/1992	NED 05/05/2006	167	167	-	-	-
7	12	M	26/10/92	NED 02/03/2004	137	137	-	-	-
8	24	M	21/06/93	AWD 14/12/1995	30	23	-	23	-
9	18	M	27/03/95	NED 13/01/1998	34	34	-	-	-
10	8	F	05/04/95	DOD 07/10/2001	78	35	-	35	78
11	17	M	19/04/95	AWD 30/04/1998	36	5	-	5	-
12	25	F	24/05/95	AWD 09/12/1997	31	22	22	22	-
13	17	M	20/11/95	DOD 03/12/1998	37	7	-	7	37
14	7	F	27/11/95	AWD 12/04/2006	101	2	2	5	-
15	18	M	15/01/96	NED 08/10/2008	105	105	-	-	-
16	14	F	14/02/96	NED 02/03/1999	37	37	-	-	-
17	16	F	15/04/96	AWD 18/05/2000	49	32	-	32	-
18	18	F	22/07/96	NED 29/04/2003	81	81	-	-	-
19	5	M	07/05/97	NED 20/04/2000	35	21	21	-	-
20	22	M	12/05/97	DOD 10/10/2001	53	26	-	26	53
21	19	F	11/02/98	NED 13/02/2007	108	108	-	-	-
22	22	F	10/06/98	NED 17/09/2008	123	123	-	-	-
23	13	F	03/08/98	NED 26/03/2002	43	43	-	-	-
24	24	M	02/09/98	NED 23/09/2008	120	24	24	-	-
25	12	M	11/11/98	NED 16/04/2008	113	113	-	-	-
26	28	M	01/12/99	AWD 08/01/2008	97	46	62	46	-
27	12	F	27/09/01	AWD 29/07/2008	82	25	25	59	-
28	17	M	16/09/02	NED 21/05/2008	68	68	-	-	-
29	19	M	02/10/03	NED 05/12/2007	50	50	-	-	-
30	15	F	24/11/03	NED 10/04/2007	41	41	-	-	-
31	14	M	27/05/04	AWD 20/05/2008	48	29	-	29	-
32	19	M	14/06/04	NED 19/08/2008	50	50	-	-	-
33	19	M	06/07/04	NED 15/07/2008	48	48	-	-	-
34	21	M	17/09/04	DOD 30/11/2008	50	10	10	20	50
35	22	F	08/11/04	NED 20/12/2008	49	49	-	-	-
36	24	F	18/11/04	NED 07/11/2007	36	36	-	-	-
37	14	F	13/01/05	NED 23/09/2008	44	44	-	-	-
38	13	M	04/04/05	NED 24/07/2007	27	27	-	-	-
39	11	F	25/04/05	NED 08/10/2008	42	42	-	-	-
40	14	F	05/05/05	NED 11/09/2007	28	28	-	-	-
41	8	M	29/08/05	NED 11/03/2008	31	31	-	-	-
42	13	M	03/10/05	NED 15/07/2008	33	33	-	-	-
43	16	M	13/10/05	NED 25/06/2008	32	32	-	-	-
44	15	M	26/01/06	NED 13/08/2008	31	31	-	-	-
45	13	M	03/04/06	NED 16/07/2008	27	27	-	-	-
46	16	M	15/05/06	NED 16/06/2008	25	25	-	-	-
47	9	M	09/06/06	NED 01/09/2008	27	27	-	-	-
48	16	M	13/07/06	NED 01/09/2008	26	26	-	-	-
49	13	F	05/10/06	NED 23/04/2009	30	30	-	-	-
50	10	M	09/10/06	NED 23/02/2009	28	28	-	-	-
Total: 50	Av.:15,96	31M - 19F	-	-	Av.:60,6	-	10/50 (20%)	15/50 (30%)	7/50 (14%)


OS: overall survival (months); DFS: disease-free survival (months); DOD: dead of disease; NED: no evidence of disease.

Age at diagnosis ranged from 5 to 28 years, averaging 15,96. Mean follow-up was 60,58 months (25-167).

Overall survival was 86%, disease-free survival was 70% and local recurrence rate was 20% in this study.

The remaining variables are described in the following table. (Table 2)

**Table 2:** Descriptive patients demographic data.

**Variable**	**Frequency**	**%**
Gender		
Male	31	62%
Female	19	38%
Anatomic Site		
Upper limb	8	18%
Lower Limb	42	84%
Type of Surgery		
Limb-sparing	43	86%
Amputation	7	14%
Microscopic surgical margin		
Negative	44	88%
Positive	6	12%
Tumor size		
< 8cm	13	26%
> 8cm	37	74%
Post chemotherapy (CT) necrosis		
< 50%	27	54%
> 50%	17	34%
Do not apply	6	12%
Local recurrence		
No	40	80%
Yes	10	20%
Distant metastasis		
No	35	70%
Yes	15	30%
Final oncologic status		
No evidence of disease	35	70%
Alive with disease	9	18%
Dead of disease	6	12%
Total	50	100%

### Histologic analysis

Slides were studied by two separate pathologists with extensive experience in musculoskeletal oncology (CRGCMO and RZF).

Osteosarcoma diagnosis was confirmed by both pathologists through hematoxilin-eosin stained slides.

All analized tissue was obtained from biopsy or resection specimens, all neither submitted to chemotherapy nor radiation therapy. Each case was studied and classified by the two pathologists, in a blind fashion regarding patients identity and clinical condition.

### Immunohistochemical analysis

Histologic sections (4µm thick) from paraffin-embedded biopsy specimens were submitted to the immunohistochemical study. The following antibody was used:

Anti-human mouse monoclonal antibody (Dako Corporation, Carpinteria CA, USA, clone VG1, isotype IgG1, kappa, VEGF isoforms 121, 165 e 189), with 1:100 dilution.

Specimens were submitted to antigenic recuperation using heat in a pressure cook. The above mentioned antibody was used in previously sylanized slides (3-aminopropyltriethoxysilane, Sigma Chemical CO, EUA, code A3 648) and left in 60°C for 24 hours, for better adhesion to the cuts. The method used was the streptavidin-biotin-peroxidase. DAKO StreptABComplex/HRP kit (estreptavidin-biotin-peroxidase) and LSAB were used in the detection reaction, as the following description:

Incubation with the primary antibody in the previously established dilution, in TBS, for an hour, at 37°C;

Washout in TBS and incubation for 5 minutes;

Incubation with the secondary antibody for 30 minutes at ambient temperature washout with TBS;

Incubation with estreptavidin-biotin-peroxidase complex for 30 minutes at ambient temperature;

Washout and subsequent posterior incubação com TBS for 5 minutes;

Incubation with DAB substract (6mg of 3,3-diaminobenzidine tetrahidrochloride in 10ml of 0,05 M TBS, pH 7.6, and 0,1ml of 3% H_2_O_2_) for 5 minutes;

Distilled water washout;

Counter-coloration with Harrys hematoxilin;

Glicerinated jelly mounting.

Positivity of the reaction was seen by the brown color, seen in 200X optic microscopy, representing the antibody-antigen formed complex. The most representative field was selected (with the highest expression). The positive cells in the selected field were counted and their percentage over the total number of cells in that field was calculated. The average percentage of the two evaluations (two pathologists) was considered for statistical purposes.

VEGF expression was classified in two groups, according to the criteria used by Kaya *et al*.^3^:

Low VEGF expression (<30% of tumor cells), named Group 1;

High VEGF expression (>30% of tumor cells), named Group 2.

### Statistical analysis

Kaplan-Meier curves regarding overall survival, disease-free survival and local recurrence were made. Low and high expression groups were compared used the log-rank test.

In order to assess the association between the variables and VEGF expression, Fisher's exact test was used.Significance level was established at 5%.

## RESULTS

**Figure 1:**
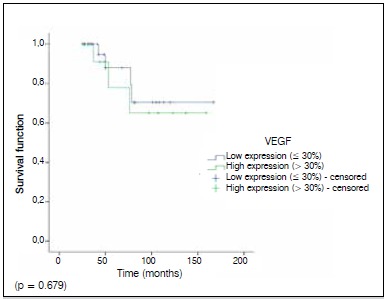
Kaplan-Meier overall survival curves for groups 1 (low VEGF expression) and 2 (high VEGF expression).

**Figure 2:**
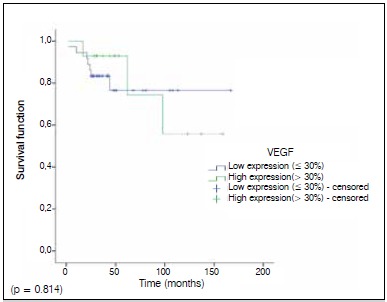
Kaplan-Meier local recurrence curves for groups 1 (low VEGF expression) and 2 (high VEGF expression).

**Figure 3:**
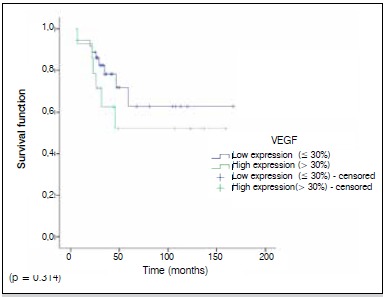
Kaplan-Meier disease free survival curves for groups 1 (low VEGF expression) and 2 (high VEGF expression).

There was no difference in the overall survival between both groups (low and high VEGF expression). For both groups it ranged between 65 and 70% at final follow-up. Figure 1 depicts the Kaplan-Meier overall survival curves for groups 1 (low VEGF expression) and 2 (high VEGF expression). There was also no difference in the recurrence-free survival: both groups had aproximately 80% at final follow-up. Figure 2

shows the Kaplan-Meier local recurrence curves for groups 1 (low VEGF expression) and 2 (high VEGF expression). The disease-free survival in both groups was also statistically similar, as shown in Figure 3. None of the variables studied were significantly related to the VEGF expression. All results are summarized in Table 3.

**Table 3:** Results.

	**VEGF expression**		
	≤** 30%**	**> 30%**	**p**
**Age**			0,056
≤ 20	32	9	
> 20	4	5	
**Gender**			0,55
Male	22	9	
Female	14	5	
**Anatomical site**			0,14
Upper limb	4	4	
Lower limb	32	10	
**Type of surgery**			0,084
Limb-sparing	29	14	
Amputation	7	0	
**Surgical margin**			0,455
Negative	31	13	
Positive	5	1	
**Tumor size**			0,264
≤ 8cm	8	5	
> 8cm	28	9	
**Post CT necrosis** **(6 patients (12%) did not receive preoperative CT)**			0,528
≤ 50%	18	9	
> 50%	12	5	
**Local recurrence**			0,717
No	29	11	
Yes	7	3	
**Distant metastasis**			0,185
No	27	8	
Yes	9	6	
**Death**			0,384
No	32	11	
Yes	4	3	

## DISCUSSION

Angiogenesis studies trying to correlate VEGF expression and osteosarcoma patients' survival rates are relatively recent. Lee *et al.*,^2^ in 1999, were the first to publish an association between high VEGF expression and bad prognosis for osteosarcoma patients. Since then, many other studies tried to establish a valid association.^2^^-^ 23 (Table 4)

**Table 4:** Literature review on VEGF expression in osteosarcoma biopsy tissue. Oncologic variables: local recurrence, overall survival and disease-free survival.

**Author and** ** publication year**	**Sample**	**Local recurrence**	**Overall survival**	**Disease-free survival**	**Tissue**	**Correlation with** **bad prognosis**
Lee *et al.* ^2^, 1999	30	20% (6)	37% (11)	30% (9)	Biopsy	Positive
Kaya *et al.* ^3^, 2000	27	ND	55% (15)	44% (12)	Biopsy	Positive
Sulzbacher *et al.* ^4^, 2002	57	5% (3)	77% (44)	65% (37)	Biopsy	Negative
Jung *et al.* ^5^, 2005	25	ND	80% (20)	68% (17)	Biopsy	Positive
Ek *et al.* ^6^, 2006	25	ND	84% (21)	72% (18)	Biopsy	Negative
Ek *et al.* ^7^, 2006	11	0% (0)	54% (6)	45% (5)	Biopsy	Negative
Oda *et al.* ^8^, 2006	30	ND	27% (8)	23% (7)	Biopsy	Positive
Mizobuchi *et al.* ^9^, 2008	48	ND	ND	ND	Biopsy	Negative
Huang *et al.* ^10^, 2008	38	ND	ND	ND	Biopsy	Positive
Bajpai *et al.* ^11^, 2009	31	ND	ND	ND	Biopsy + Resection	Positive
Zhou *et al.* ^12^, 2009	65	26% (17)	26% (17)	ND	Biopsy	Positive
Abdeen *et al.* ^13^, 2009	48	ND	71% (34)	67% (32)	Biopsy + Resection + Metastasis	Negative
Marinho^14^, 2009 (DT)	50	ND	50% (25)	44% (22)	Biopsy	Positive
Rossi *et al.* ^15^, 2010	16	31% (5)	69% (11)	56% (9)	Biopsy + Resection	Positive
Lin *et al.* ^16^, 2011	56	20% (11)	45% (25)	45% (25)	Biopsy	Positive
Lugowska *et al.* ^17^, 2011	91	ND	73% (66)	56% (51)	Biopsy	Positive
Qu *et al.* ^18^, 2012 (MA)	387	ND	ND	ND	Biopsy	Positive
Rastogi *et al.* ^19^, 2012	40	ND	ND	ND	Serum + biopsy	Negative
Lammli *et al.* ^20^, 2012	54	17% (9)	ND	59% (32)	Biopsy	Positive
Chen *et al.* ^21^, 2013 (SR)	559	ND	ND	ND	Biopsy	Positive
Becker *et al.* ^22^, 2013	27	ND	67% (8)	40% (11)	Biopsy	Negative
Yu *et al.* ^23^ , 2014 (MA)	323	ND	ND	ND	Biopsy	Positive
Present study	50	20%(10)	86%(43)	70% (35)	Biopsy	Negative

ND = not described; DT = Doctorate thesis (unpublished); MA = meta-analysis; SR = Systematic review.

Regarding the local recurrence rate, we had ten cases (20%). Kaplan-Meier curves comparing the two groups did not obtain statistical significance between the VEGF expression and local recurrence (p=0.814). When analyzing by the Fisher's Exact test, we observed that among the ten locally recurred cases, three showed high VEGF expression (30%). In the other 40 patients that did not experience local recurrence, eleven showed high VEGF expression (27%) (p=0.717).

When comparing with the literature, we see that most of the similar studies do not even mention local recurrence as a variable.^5^^,^ 9 ^,^ 13 ^,^ 14 ^,^ 17 ^,^ 22 In the few studies that mention local recurrence, numbers obtained were similar to the present study. Lee *et al.* 2 and Lin *et al.* 16 reported the same 20% of local recurrence of the present series. Zhou *et al.*,^12^ presented 26% of local recurrence, and Rossi *et al.*,^15^ reported 31%. Only two studies reported unusually low recurrence rates: Sulzbacher *et al*.,^4^ with 5% (3/57), and Ek *et al.*,^7^ with no case in 11 patients.

When considering disease-free survival, the present series showed a 70% rate (35/50). As in our study only extremity cases were selected, all 10 locally recurrent cases were managed with amputation. Thus, no patient alive with disease had local recurrence: all had metastatic disease.

Kaplan-Meier curves of the two groups regarding disease-free survival did not show any difference regarding VEGF expression (p=0.314). However, when we compare the patients with metastasis with the disease-free patients, we observe a slight trend to metastatic patients present higher VEGF expression (p=0.185). All metastatic cases had pulmonary metastasis, being one case also with soft tissue metastasis. Four of the 15 patients with pulmonary metastasis were submitted to metastasectomies, one of them to three consecutive procedures.

The staining results of VEGF were classified into negative (<30%) or positive (>30%), the same criteria used by Kaya *et al.* 3 However, there is no consensus on which threshold to use. Huang *et al*.,^10^ Rossi *et al*.,^15^ Łudowska *et al.* 17 and Rastogi *et al.*,^19^ for instance, used 50% for the threshold value. Qu *et al.* 18 recommend 25% as a VEGF positive cutt-off value. Lammli *et al.* 20 used an even lower cut-off value of 20%.

Rastogi *et al.* 19 found a significant correlation between high serum VEGF and a high percentage of cells showing VEGF expression (p<0.001), when using 50% as a cut-off value. In the study, the patients who developed pulmonary metastasis had a higher baseline mean serum VEGF (p<0,001). The study failed, however, to show correlation between high VEGF serum values and all the others variables including overall survival and tumor staging.

The present study had a high disease-free survival of 70%, while in the literature it ranged from 23% to 72%. One explanation might be that we selected only patients with localized disease, and the majority of the studies with lower disease-free survival did not exclude the patients with metastasis at diagnosis.

Finally, our study showed 86% of overall survival. From the 50 initial cases, 15 developed distant metastasis (30%) and seven died of the disease (14%). We did not obtain statistically significant correlation between the VEGF expression in the biopsy tissue and the occurrence of death in the Kaplan-Meier curves analysis (p=0.679). When observing the Fisher's exact test, we detect a small trend to patinets that died had higher VEGF expression. But no statistical significance was obtained (p=0.384).

When comparing the present study with the literature, we see great variations in survival rates. Rates ranged from 26%^12^ to 84%.^6^ There are explanations, though, for this fact.

In the study from Zhou *et al.*,^12^ for instance, 28 of the 65 patients (43%) entered the study with metastatic disease. Oda *et al.* 8 included only patients with pulmonary metastasis, on the contrary to the present study, that selected only nonmetastatic cases. In the thesis from Marinho,^14^ 22 of the 50 patients were metastatic at diagnosis. Ługowska *et al.*,^17^ who also selected only non-metastatic patients, also had a relatively high overall survival rate of 73%. These data should be taken in account when comparing the results of each study.

When we take only the studies that used biopsy tissue, we see that, among the 18 (excluding the two meta-analysis and the systematic review) studies, 12 show a positive correlation between high VEGF expression and poor prognosis, opposed to the other six studies. There was also one study that showed no correlation, but the VEGF was measured in the serum.^19^ The present study did not show significant correlation between the VEGF expression and poor prognosis. Therefore, we have 12 studies that propose a correlation between high VEGF expression and poor prognosis, and eight studies not correlating these variables.

Two meta-analysis^18^^,^ 23 and one systematic review^21^ confirmed the inverse association between the levels of VEGF and survival. The meta-analysis by Qu *et al* 18 found a positive association of elevated VEGF with the death of patients in the first five years after diagnosis (2.85-fold higher 5-year mortality). This meta-analysis also confirmed through univariate analysis that the higher stage of osteosarcoma, the patients from less developed areas, the lower percentage of osteoblastic histotype, the higher percentage of osteosarcoma located to femur and tibia and the more share of patients underwent neochemotherapy were risk factors of patients' survival. The percentage of VEGF expression, however, meant little. They stated that there is a small inverse relationship between VEGF expression level and the 5-year survival of osteosarcoma patients.

The meta-analysis by Yu *et al*.^23^ also found a inverse relationship between the levels of VEGF and prognosis. Interestingly, when grouped according to geographic settings of individual studies, the combined hazard ratio of Asian studies and non-Asian studies were 2.7 (95% CI: 1.35 - 3.39) and 1.51 (95% CI: 0.89-2.14), respectively, indicating that VEGF is an indicator of poor prognosis of osteosarcoma in Asian patients but not in non-Asian patients. This might be another reason for the present study not having reached a significant association between VEGF levels and prognosis, as our population is of non-Asian patients.

Although the present study is among a minority, a global view allows us to assume that VEGF expression seems to be a prognostic factor regarding survival in osteosarcoma patients. 

## CONCLUSION

VEGF expression in biopsy tissue was not a prognostic factor for nonmetastatic osteosarcoma of the extremities in this study.
